# Supplementary material for: HIVprotI: an integrated web based platform for prediction and design of HIV proteins inhibitors
Source: J Cheminform. 2018 Mar 9;10:12. doi: 10.1186/s13321-018-0266-y (PMC5845081; doi:10.1186/s13321-018-0266-y)
Supplement: Supplementary file 2 — Additional file 2. Source code of HIVProtI web server. [file 13321_2018_266_MOESM2_ESM.zip › HIVprotI_Source-code/hivprotI/weka-3-6-6/doc/weka/attributeSelection/AttributeEvaluator.html]

AttributeEvaluator


---


|  |  |  |
| --- | --- | --- |
| **Overview** \| **Package** \| **Class** \| **Tree** \| **Deprecated** \| **Index** \| **Help** \| **Weka's home** | |  |
| **PREV CLASS**   **NEXT CLASS** | **FRAMES**    **NO FRAMES**     **All Classes** |  |
| SUMMARY: NESTED \| FIELD \| CONSTR \| METHOD | DETAIL: FIELD \| CONSTR \| METHOD |  |


---


## weka.attributeSelection Interface AttributeEvaluator

**All Known Implementing Classes:**: ChiSquaredAttributeEval, CostSensitiveAttributeEval, FilteredAttributeEval, GainRatioAttributeEval, InfoGainAttributeEval, LatentSemanticAnalysis, OneRAttributeEval, PrincipalComponents, ReliefFAttributeEval, SVMAttributeEval, SymmetricalUncertAttributeEval, UnsupervisedAttributeEvaluator

---

``` public interface AttributeEvaluator ```

Interface for classes that evaluate attributes individually.

**Version:**
:   $Revision: 1.11 $

**Author:**
:   Mark Hall (mhall@cs.waikato.ac.nz)

---

| **Method Summary** | |
| --- | --- |
| `double` | `evaluateAttribute(int attribute)`             evaluates an individual attribute |

**Method Detail**

### evaluateAttribute

```
double evaluateAttribute(int attribute)
                         throws java.lang.Exception
```

:   evaluates an individual attribute

    :   **Parameters:**: `attribute` - the index of the attribute to be evaluated **Returns:**: the "merit" of the attribute **Throws:**: `java.lang.Exception` - if the attribute could not be evaluated


---


|  |  |  |
| --- | --- | --- |
| **Overview** \| **Package** \| **Class** \| **Tree** \| **Deprecated** \| **Index** \| **Help** \| **Weka's home** | |  |
| **PREV CLASS**   **NEXT CLASS** | **FRAMES**    **NO FRAMES**     **All Classes** |  |
| SUMMARY: NESTED \| FIELD \| CONSTR \| METHOD | DETAIL: FIELD \| CONSTR \| METHOD |  |


---
